# Multicomponent DNA vaccine-encoding *Toxoplasma gondii* GRA1 and SAG1 primes: anti-*Toxoplasma* immune response in mice

**DOI:** 10.1007/s00436-012-3047-y

**Published:** 2012-07-27

**Authors:** Xiao-Nan Wu, Jie Lin, Xu Lin, Jie Chen, Zhong-Long Chen, Jian-Yin Lin

**Affiliations:** 1 Key Laboratory of Ministry of Education for Gastrointestinal Cancer, Research Center of Molecular Medicine, Fujian Medical University, Fuzhou, 350004 People’s Republic of China; 2 Public Health School, Fujian Medical University, Fuzhou, 350004 People’s Republic of China; 3 Fujian Center for Disease Control and Prevention, Fuzhou, 350001 People’s Republic of China

## Abstract

A multicomponent DNA vaccine, encoding *Toxoplasma gondii* GRA1 and SAG1, was constructed and tested for its ability to confer protection. BALB/c mice were challenged with tachyzoites of the virulent *T*. *gondii* RH strain at 4 weeks following the last immunization, and immune responses and survival times were observed. The results show that vaccination by the multicomponent vaccine prolonged survival of mice challenged with the *T*. *gondii* RH strain (from average 4.50 ± 0.22 to 7.60 ± 0.74 days); induced high levels of IgG antibody (from 0.252 ± 0.080 to 0.790 ± 0.083), IFN-gamma (from 598.74 ± 67.50 to 853.77 ± 66.74 pg/ml), and IL-2 (from 89.44 ± 10.66 to 192.24 ± 19.90 pg/ml); changed the CD4^+^/CD8^+^ lymphocyte ratio (from 1.81 ± 0.14 to 1.09 ± 0.19); and stimulated NK cell-killing activity (from 46.81 ± 3.96 to 64.15 ± 7.71 %). These findings demonstrate that a multicomponent DNA vaccine, encoding GRA1 and SAG1, primes a strong humoral and cellular immune response and enhances protection against *T*. *gondii* challenge. The new, combined DNA vaccine provides another means to combat *T*. *gondii* infection.

## Introduction

*Toxoplasma gondii* is an intracellular protozoan parasite that causes significant morbidity and mortality in congenitally infected and immunocompromised individuals. In humans, this relatively benign infection may reactivate under conditions of immunosuppression, such as in HIV-infected individuals and cancer patients, resulting in *Toxoplasma* encephalitis and other complications (Calabrese et al. 2008; Kato et al. 2005; Scorza et al. 2003). *T*. *gondii* infection acquired by pregnant women and its transmission to the fetus continue to be the cause of tragic yet preventable disease (Montoya and Remington 2008), and it is associated with transplacental infection. In veterinary medicine, *T*. *gondii* infection has economic impact by inducing abortion and neonatal loss in sheep and goats and is a source of transmission to humans (Dubey 1990). Therefore, the development of an effective vaccine against *T*. *gondii* would be of great value to both human and veterinary medicine.

Recently, there have been efforts to develop anti-*T*. *gondii* vaccines (Bhopale 2003). Among the new approaches, DNA vaccines have become a focus since they elicit potent, long-lasting humoral and cell-mediated immunity (Alarcon et al. 1999). Membrane-associated surface antigens (SAG1 and SAG2), secreted dense-granule proteins (GRA1, GRA2, GRA4, and GRA7), rhoptry proteins (ROP1 and ROP2), and micronemal proteins (MIC1, MIC2, MIC3, MIC4, and MIC8) are all putative *T*. *gondii* vaccine candidates (Dautu et al. 2007; Fang et al. 2009; Jongert et al. 2007; Li et al. 2011; Liu et al. 2010, 2009; Wang et al. 2009; Xue et al. 2008; Zhang et al. 2007). These antigens have shown certain protection, increased survival time of animals, and a reduced number of brain cysts in rodents. Also, employment of different forms of adjuvant was evaluated and compared the effects on the immune response stimulated by DNA vaccine (Khosroshahi et al. 2012).

SAG1 is the best-characterized candidate vaccine. Previous studies have shown that DNA vaccines with SAG1 induce both humoral and cellular immune responses and partial protection against *T*. *gondii* (Couper et al. 2003; Fachado et al. 2003; Liu et al. 2009; Mevelec et al. 2005; Xue et al. 2008; Zhang et al. 2007). Dense-granule antigens (GRA), secreted in abundance, are major components of both the vacuole-surrounding tachyzoites and the cyst wall-surrounding slower growing bradyzoites (Cesbron-Delauw 1994). Therefore, the GRAs may be important protective antigens. Among the GRAs, GRA1, a product of *T*. *gondii* tachyzoites and bradyzoites, is a promising candidate. A GRA1 DNA vaccine can prime an anti-*Toxoplasma* immune response (Bivas-Benita et al. 2003; Jongert et al. 2007; Vercammen et al. 2000). In addition, native protein encoded by GRA1 is a type of Ca^2+^-binding protein that functions to activate or stabilize the reticulum structure and may also function as a Ca^2+^ buffer (Lin et al. 2010). GRA1 and SAG1 possess distinct antigenicity, and their expression spans different stages of *T*. *gondii* development. Therefore, the objectives of this study were to construct eukaryotic expression plasmids, pVAX1-GRA1 and pVAX1-SAG1, and to evaluate the immune response and protective effect of a combined DNA vaccine in BALB/c mice against challenge with a highly lethal *T*. *gondii* RH strain. The results show that a multicomponent DNA vaccine, encoding *T*. *gondii* GRA1 and SAG1, primed a strong humoral and cellular immune response and enhanced protection against *T*. *gondii* challenge.

## Materials and methods

### Cell lines, plasmids, mice, and reagents

Raw264.7 cell, a murine macrophage cell line, was obtained from the Shanghai Cell Biological Institute (Shanghai, China). Eukaryotic expression vector pVAX1 was purchased from Invitrogen (Carlsbad, CA, USA). SPF grade BALB/c mice were purchased from Shanghai SLAC Laboratory Animal Co., Ltd. (Shanghai, China).

Taq DNA polymerase (high fidelity) was purchased from Stratagene (Santa Clara, CA, USA). Restriction enzymes (*Eco*R I and *Kpn* I), calf intestine alkaline phosphatase, and T4 DNA Ligase were purchased from New England Biolabs (Beverly MA, USA). Endonuclease-Free Plasmid Mega kit was from QIAGEN (Hilden, Germany). Liposome (Lipofectamine 2000), and kanamycin were obtained from Invitrogen. Mouse anti-*T*. *gondii* SAG1 and rabbit anti-*T*. *gondii* trophozoite antibodies were from Biodesign (Saco ME, USA). 3-(4,5-Dimethylthiazol-2-yl)-2,5-diphenyltetrazolium (MTT) was obtained from Sigma (St. Louis, MO, USA). Fluorescein isothiocyanate (FITC)-labeled anti-mouse CD4 antibody, polyethylene (PE)-labeled anti-CD8 antibody was from Beckman Coulter (Shanghai, China). Detection kits for mouse IFN-γ, IL-2, and IL-4 cytokines (OptEIA™) were from BD Bioscience Corp (Shanghai, China).

### Primers

The following primers were used: GRA1VAXF, 5′CGGGGTACCATGGTGCGTGTGAGCGCTATTG (*Kpn* I); GRA1VAXR, 5′ CCGGAATTCTTACTCTCTC TCTCCTGTTAGG (*Eco*R I); SAG1VAXF, 5′CGGGGTACCATGTCGGTTTCGCTGCACCAC (*Kpn* I); and SAG1VAXR, 5′ CCGGAATTCTCACGCGACACAAGCTGCG (*Eco*R I).

### Amplification of GRA1 and SAG1 genes

GRA1 or SAG1 was amplified from recombinant plasmid pCMV/myc-GRA1 or pCMV/myc-SAG1 (constructed in our previous project) templates, respectively. Briefly, samples were denatured for 5 min at 94 °C, followed by 30 cycles of denaturation (94 °C, 30 s), with annealing (55 °C, 30 s) and extension (72 °C, 1 min), followed by a final extension at 72 °C for 10 min. PCRs were performed with the primer pairs GRA1VAX F-R or SAG1VAX F-R.

### Construction of the recombinant plasmids pVAX1-GRA1 and pVAX1-SAG1 and large-scale DNA preparation

PCR products were cloned into the pVAX1 vector. Sequencing was performed on an ABI PRISM genetic analyzer in Takara Biotechnology (Dalian, China) using primers T7 (F) and BGH (R). Sequence alignments were done using BLAST with default settings (PubMed, http://blast.ncbi.nlm.nih.gov). All plasmids were propagated in *Escherichia coli* DH5-α.

The plasmids pVAX1-GRA1 and pVAX1-SAG1 were purified by Endo-Free Plasmid Mega kit prior to using for vaccination and dissolved in sterile endotoxin-free phosphate-buffered saline (PBS). Plasmid integrity was checked by agarose gel electrophoresis after digestion with appropriate restriction enzymes. The DNA concentration and purity was determined by A_260_.

### Macrophage transfection by liposome and gene expression

One day before transfection, RAW264.7 was inoculated into six well plates (Corning Incorporated, Corning, NY, USA) with 2-ml common culture medium and cultured in 5 % CO_2_ at 37 °C to 80 % confluence. Five micrograms DNA (pVAX1-GRA1, pVAX1-SAG1, or pVAX1) were diluted in 50-μl sera-free medium (10 μl liposome diluted in 40-μl serum-free medium) and mixed. DNA and liposomes were incubated for 20 min at room temperature to form DNA–liposome complexes. Transfection mixture (100 μl) was added to the macrophage culture and gently mixed. The plates were then incubated in 5 % CO_2_ at 37 °C for 48 h.

GRA1 and SAG1 expression was detected by immunohistochemistry. Briefly, RAW264.7 (transfected by pVAX1-GRA1, pVAX1-SAG1, empty control plasmid pVAX1, or nontransfected), grown on the cover slip, was fixed by prechilled 70 % alcohol for 30 min. Cells were rinsed three times with PBS for 3 min, incubated with 50 μl peroxidase-blocking solution for 10 min, and rinsed three times with PBS. Nonimmune serum was incubated for 10 min, and the surplus was discarded. Primary antibody (50 μl) was then incubated for 60 min, rinsed three times with PBS, and followed by 50 μl biotin-conjugated secondary antibody for 10 min and a rinse with PBS (three times). Complexes were visualized by incubation with 50 μl streptavidin peroxidase solution for 10 min, PBS rinse (three times), and addition of 100 μl freshly prepared DAB for 3–10 min. Following a wash by double distilled water, samples were counterstained by hematoxylin and washed by PBS. Unless noted, all incubations and washes were performed at room temperature. Samples were photographed using the Leica photo system (Solms, Germany).

### Vaccination

BALB/c mice were randomly divided into five groups of 18 each. Plasmids were diluted to 2 μg/μl in PBS. The five groups were: 50 μl PBS, 100 μg empty pVAX1 vector, 100 μg pVAX-GRA1, 100 μg pVAX1-SAG1, and 50 μg pVAX1-GRA1 + 50 μg pVAX1-SAG1, respectively. Each group received three injections (separated by 2-week intervals) in both tibias and anterior muscles. Injections were administered with a 0.3-ml syringe.

### Challenge experiments

Four weeks after the last vaccination, ten mice were selected randomly from each group and challenged by intraperitoneal injection with 10^5^ tachyzoites of *T*. *gondii* RH strain. The length of survival was recorded.

### Evaluation of the humoral immune response

IgG antibodies were monitored by ELISA. The eyes were extirpated to get sera from mice 4 weeks after the third vaccination. Ninety-six-well plates were coated with tachyzoite lysis antigen of *T*. *gondii*. Serum samples were diluted (1:100) prior to testing.

### Evaluation of the cellular immune response

ELISA was used to measure IFN-γ, IL-2, and IL-4 concentrations. The ratio of CD4^+^/CD8^+^ in spleen cells was measured by Beckman Coulter XL flow cytometry (488 nm excitation, 620 nm emission for PE, and 525 nm for FITC), using 0.5 × 10^6^ cells.

### NK cell-killing activity

Splenocytes (effector cells) obtained from mice 4 weeks after the third vaccination were adjusted to 5 × 10^6^/ml in RPMI 1640 with 5 % fetal bovine serum. The eugenic Yac-1 cells (target cells) were adjusted to 2 × 10^5^/ml in RPMI 1640. Effector and target cells were plated in 100 μl 96-well flat-bottomed plates at E/T ratios of 25:1. Effector and target cells, alone in RPMI 1640, served as controls. All assays were done in triplicate. After a 44-h incubation at 5 % CO_2_ and 37 °C, 20 μl MTT was added to each well and further incubated at 5 % CO_2_, 37 °C for 4 h. Excess supernatant was discarded, and 100 μl 100 % DMSO was added to each well, and mixed for 10 min, and the A_570_ was measured. Cytotoxicity (in percent) = [(experimental − effector spontaneous) / target spontaneous] × 100.

## Results

### The construction of DNA vaccines and identification and expression in macrophages

**Fig. 1:**
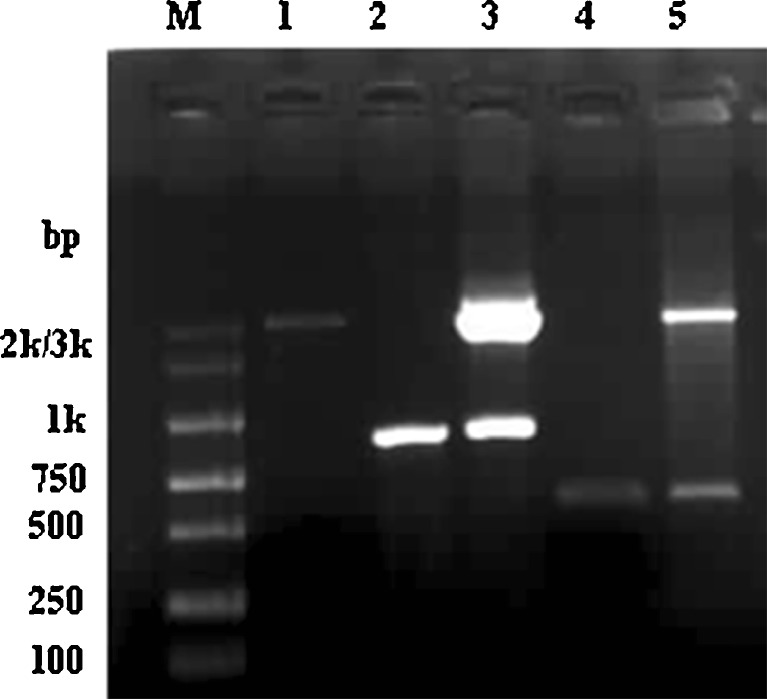
Characterization of recombinant plasmids pVAX1-GRA1 and pVAX1-SAG1 by PCR and restriction enzyme digestion. *M* DNA marker, *1* pVAX1, *2* amplified fragment of SAG1 gene, *3* recombinant plasmid pVAX1-SAG1 digested by *Kpn* I and *Eco*R I, *4* amplified fragment of GRA1 gene, *5* recombinant plasmid pVAX1-GRA1 digested by *Kpn* I and *Eco*R I

A 573-bp PCR product, corresponding to the GRA1 coding sequence, was generated, digested with the appropriate restriction enzymes, and cloned into the expression vector pVAX1, generating pVAX1-GRA1. A 960-bp PCR product for SAG1 was created the same way as for GRA1, generating pVAX1-SAG1. Sequence alignment for both GRA1 and SAG1 by PubMed verified that the sequence was correct. Figure 1 shows the identification of recombinant plasmid pVAX1-GRA1, pVAX1-SAG1 by PCR, and restriction enzyme digestion.

**Fig. 2:**
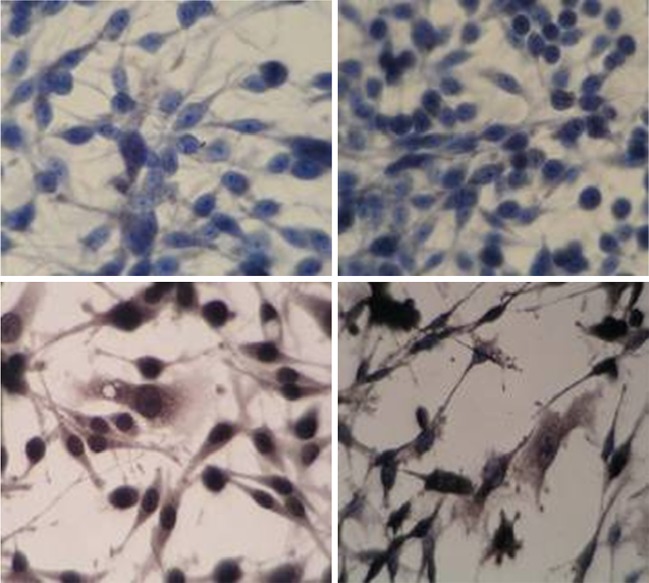
Expression of GRA1/SAG1 antigen in mouse macrophage (immunocytochemistry, ×400). *Upper left* RAW264.7, *upper right* RAW264.7 transfected with pVAX1, *lower left* RAW264.7 transfected with pVAX1-SAG1, *lower right* RAW264.7 transfected with pVAX1-GRA1

To determine whether the *T*. *gondii* GRA1 and SAG1 proteins were expressed in RAW264.7, following transfection with pVAX1-GRA1, pVAX1-SAG1, or an empty vector, expression was tested by immunohistochemistry. Brown-stained particles were found in the cytoplasm that had been transfected by pVAX1-GRA1 or pVAX1-SAG1. No stain was observed in cells transfected with control vector pVAX1 (Fig. 2).

### Protective effect of GRA1 and SAG1 DNA vaccine in BALB/c mice challenged with *T*. *gondii*

**Fig. 3:**
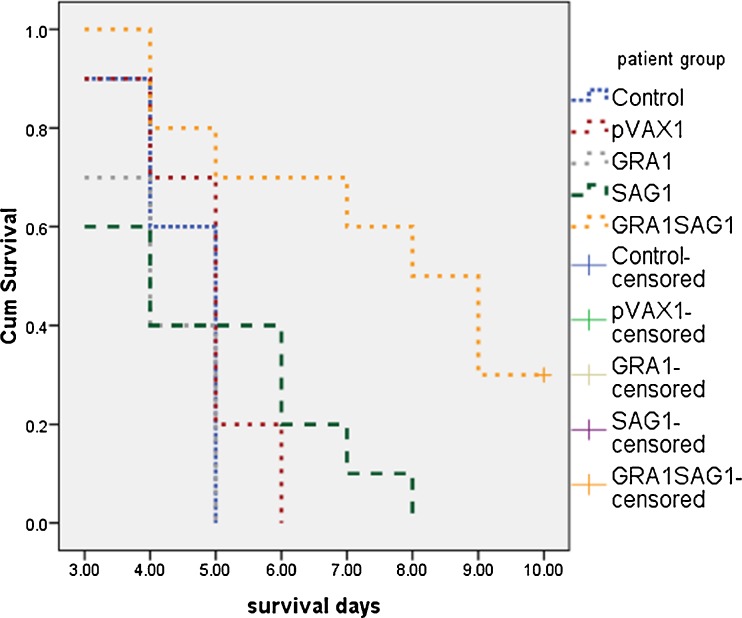
Survival curves of BALB/c mice injected with 50 μl PBS or immunized with 100 μg empty pVAX1, 100 μg pVAX-GRA1, 100 μg pVAX1-SAG1, 50 μg pVAX1-GRA1 + 50 μg pVAX1-SAG1, respectively, and challenged with 10^5^ tachyzoites of *T*. *gondii* RH strain 4 weeks after last vaccination. Each group contained ten mice. Survival data were recorded until the end of the observation period

To test whether the DNA vaccine, encoding *T*. *gondii* GRA1 and SAG1, could protect against a lethal *T*. *gondii* infection, BALB/c mice were immunized with 100 μg pVAX1 empty vector, 100 μg pVAX-GRA1, 100 μg pVAX1-SAG1, or 50 μg pVAX1-GRA1 + 50 μg pVAX1-SAG1, respectively. The survival time is shown in Fig. 3. Control mice died beginning on day 3, and all mice had died by day 5. The mice lived on average, just 4.50 ± 0.22 days, following infection. Mice immunized with pVAX1 died beginning on day 3, and all had died by day 6, with an average of 4.80 ± 0.29 days. The mice of the pVAX1-GRA1 group died beginning on day 3, and all had died by day 5, with an average of 4.10 ± 0.28 days. The mice of the pVAX1-SAG1 group also began to die from the third day on, and all had died by day 8, (average = 4.70 ± 0.60 days). Mice immunized with pVAX1-GRA1-SAG1 began to die beginning on day 4; however, three mice were still alive on day 10 (average = 7.60 ± 0.74 days). This last group lived longer than the other groups (*P* < 0.05). The statistical significance between different groups was demonstrated by survival analysis (log-rank statistic, Table 1). The data indicate that multicomponent DNA vaccines, encoding *T*. *gondii* GRA1 and SAG1, can increase the survival time of mice challenged with *T*. *gondii* RH strain.

**Table 1:** Results of log-rank statistics for survival time of BALB/c mice challenged with 10^5^ tachyzoites of *T*. *gondii* RH strain 4 weeks after last vaccination (*N* = 10)

Group	Control	pVAX1	pVAX1-GRA1	pVAX1-SAG1	pVAX1-GRA1 + pVAX1-SAG1
Control	–	1.07 (0.3000)	1.04 (0.3090)	0.33 (0.5680)	7.97 (0.0048)
pVAX1	1.07 (0.3000)	–	2.98 (0.0840)	0.12 (0.7263)	8.11 (0.0044)
pVAX1-GRA1	1.04 (0.3090)	2.98 (0.0840)	–	1.26 (0.2620)	9.97 (0.0016)
pVAX1-SAG1	0.33 (0.5680)	0.12 (0.7263)	1.26 (0.2620)	–	8.10 (0.0044)
pVAX1-GRA1 + pVAX1-SAG1	7.97 (0.0048)	8.11 (0.0044)	9.97 (0.0016)	8.10 (0.0044)	–

The values in the table are chi-square values tested by log-rank statistic of pairwise comparisons. The values in parentheses are significance values

### Anti-*Toxoplasma* IgG levels in BALB/c mice

**Fig. 4:**
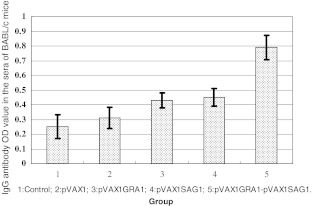
Levels of IgG antibody in sera from BALB/c mice immunized with single- or two-gene DNA vaccines, empty vector, or PBS (control group) as measured by ELISA 4 weeks after last vaccination. Each group was composed of eight mice

To evaluate the immunogenicity of the GRA1 and SAG1 DNA vaccines, BALB/c mice were immunized with empty vector or single- or two-gene vaccines. Mice were bled, and anti-*Toxoplasma* IgG levels were determined by ELISA 4 weeks following the last vaccination (Fig. 4). The anti-*Toxoplasma* IgG optical density (OD) value was 0.790 ± 0.083 in the pVAX1-GRA1 + pVAX1-SAG1 group, while the values were 0.430 ± 0.052 and 0.451 ± 0.060 in pVAX1-GRA1 and pVAX1-SAG1 groups, respectively. The OD values were 0.310 ± 0.071 in the control vector group and 0.252 ± 0.080 for the PBS control. Using ANOVA statistical analysis, the OD values in the pVAX1-GRA1 + pVAX1-SAG1, pVAX1-GRA1, and pVAX1-SAG1 were significantly higher than those in PBS control group (*P* < 0.05). The values for the pVAX1-GRA1 + pVAX1-SAG1 were higher than those for pVAX1-SAG1 and pVAX1-GRA1 (*P* < 0.05). These results indicate that a multicomponent DNA vaccine, encoding *T*. *gondii* GRA1 and SAG1, induces a strong anti-*T*. *gondii* IgG antibody response.

### Production of IFN-γ, IL-2, and IL-4 in sera from BALB/c mice

**Fig. 5:**
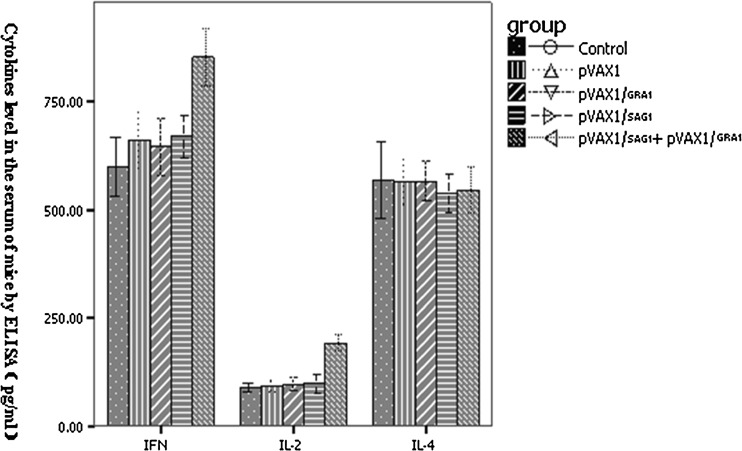
Levels of IFN-γ, IL-2, and IL-4 in sera from the BALB/c mice immunized with the single- or two-gene DNA vaccine, the empty vector, or PBS (control) as measured by ELISA 4 weeks following the last vaccination. Each group was composed of eight mice

To evaluate the role of cytokines in the cellular immune response against *T*. *gondii* infection, IFN-γ, IL-2, and IL-4 serum levels in immunized mice were measured by ELISA (Fig. 5). The IFN-γ levels in the pVAX1-GRA1 + pVAX1-SAG1 (853.77 ± 66.74 pg/ml), pVAX1 (679.63 ± 44.79 pg/ml), and pVAX1-SAG1 (669.78 ± 50.42 pg/ml) groups were higher relative to those of control group (598.74 ± 67.50 pg/ml) (*P* < 0.05). The IFN-γ levels in the pVAX1-GRA1 + pVAX1-SAG1 group were elevated relative to those of pVAX1-GRA1 and pVAX1-SAG1 (*P* < 0.05). IL-2 levels in the pVAX1-GRA1 + pVAX1-SAG1 group (192.24 ± 19.90 pg/ml) were higher than those of empty pVAX1 vector (92.77 ± 13.94 pg/ml) and PBS control (89.44 ± 10.66 pg/ml) (*P* < 0.05). In contrast, the IL-4 levels of the five groups were similar (*P* > 0.05). The data show that the DNA vaccine, encoding GRA1 and SAG1, is able to produce higher levels of IFN-γ and IL-2.

### Percent change of CD4^+^/CD8^+^ T cells in splenocytes from BALB/c mice immunized with DNA vaccines

Protection against *T*. *gondii* is dependent on both CD4^+^ and CD8^+^ cells (Li et al. 2010). To evaluate the role of CD4^+^ and CD8^+^ cells in the cellular immune response against *T*. *gondii* challenge, the percent change of CD4^+^/CD8^+^ T cells in splenocytes from immunized mice was measured by flow cytometry (Table 2). Compared with PBS control, the percent of CD4^+^ cells in the pVAX1-GRA1 + pVAX1-SAG1 (37.42 ± 4.84 %), pVAX1-SAG1 (44.32 ± 2.61 %), and pVAX1-GRA1 (45.82 ± 3.01 %) groups was reduced (*P* < 0.05). The percent of CD8^+^ cells in the pVAX1-GRA1 + pVAX1-SAG1 group (34.94 ± 5.30 %) increased relative to that of pVAX1 or PBS control (*P* < 0.05). The CD4^+^/CD8^+^ ratio of pVAX1-GRA1 + pVAX1-SAG1 (1.09 ± 0.19 %), pVAX1-SAG1 (1.56 ± 0.22 %), and pVAX1-GRA1 (1.66 ± 0.13 %) groups decreased relative to that of pVAX1 (*P* < 0.05). The CD4^+^/CD8^+^ ratio of pVAX1-GRA1 + pVAX1-SAG1 was lower than that observed for the pVAX1-GRA1 and pVAX1-SAG1 groups (*P* < 0.05).

**Table 2:** Flow cytometry analysis of T lymphocyte subsets in splenocytes from BALB/c mice 4 weeks after last vaccination (*N* = 8)

Group	T 1ymphocyte subsets		
	CD4^+^ (%)	CD8^+^ (%)	CD4^+^/CD8^+^
PBS control	48.59 ± 1.85	26.91 ± 2.12	1.81 ± 0.14
pVAX1	48.01 ± 2.32	26.35 ± 1.69	1.83 ± 0.12
pVAX1-GRA1	45.82 ± 3.01^a^	27.82 ± 3.24	1.66 ± 0.13^b^
pVAX1-SAG1	44.32 ± 2.61^a, b^	28.89 ± 4.87	1.56 ± 0.22^a, b^
pVAX1-GRA1 + pVAX1-SAG1	37.42 ± 4.84^a, b^	34.94 ± 5.30^a, b^	1.09 ± 0.19^a, b^

^a^Compared with the PBS control group, *P* < 0.05

^b^Compared with the pVAX1 group, *P* < 0.05

### NK cell-killing activity in BALB/c splenocytes from mice immunized with DNA vaccine

**Fig. 6:**
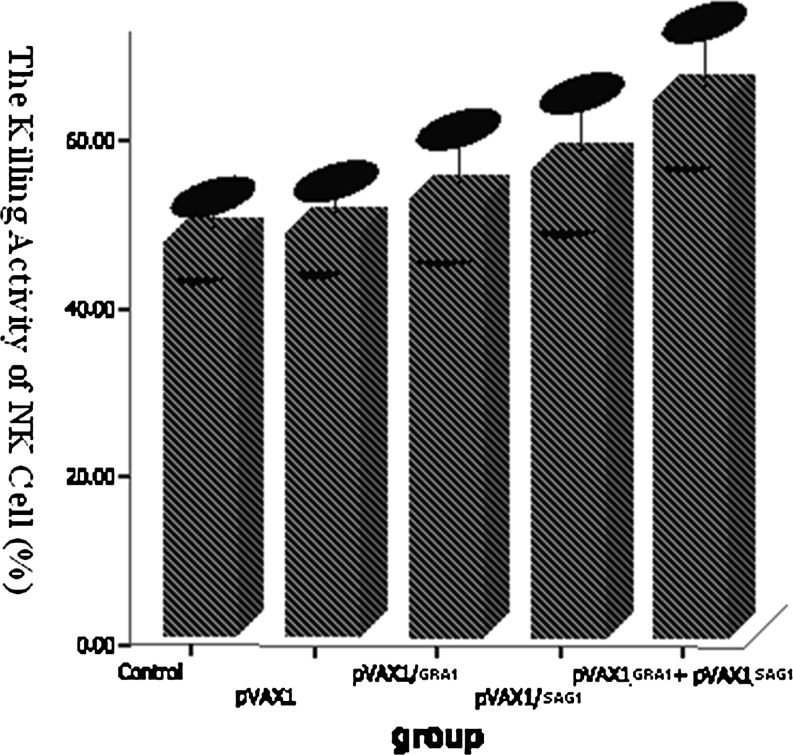
The NK cell-killing activity of splenocytes from BALB/c mice 4 weeks after the last vaccination. Each group was composed of eight mice

To test whether NK cells play an important role in the cellular immune responses induced by the GRA1 and SAG1 vaccine, the NK cell-killing activity of splenocytes was determined by MTT assays (Fig. 6). The NK cell-killing rate of splenocytes of pVAX1-GRA1 + pVAX1-SAG1, the pVAX1-GRA1, the pVAX1-SAG1, the pVAX1 empty vector, and the PBS control group was 64.15 ± 7.71, 52.03 ± 6.96, 55.95 ± 7.37, 48.13 ± 4.58, and 46.81 ± 3.96 %, respectively. ANOVA analysis indicated that the increased killing of the pVAX1-GRA1 + pVAX1-SAG1, pVAX1-GRA1, and pVAX1-SAG1 groups were statistically significant relevant to the control group (*P* < 0.05). The killing rate of the pVAX1-GRA1 + pVAX1-SAG1 NK cells was higher than that of the pVAX1-GRA1 and pVAX1-SAG1 NK cells (*P* < 0.05).

## Discussion

The potential for a multicomponent DNA vaccine, encoding *T*. *gondii* GRA1 and SAG1 to induce protective immune responses in BALB/c mice, was investigated. The results indicate that the vaccine primes humoral and cellular responses and enhances protection against *T*. *gondii* challenge.

The challenge dose may be a factor that influences the observed protection level. In this study, we sought to assess vaccine protection against a high-dose challenge (with regard to mouse) because humans are not considered to be as sensitive to *Toxoplasma* as mouse, especially at the lethal dose. The lethal dose of 10^5^ RH parasites in mouse (Fachado et al. 2003) may not be lethal if the equivalent were used in humans. Interestingly, even at this high-dose level, three mice vaccinated with the multicomponent vaccine were still alive on day 10; however, all control mice had died by day 5, and statistical significance was observed. The results suggest that combined immunization of pVAX1-GRA1 and pVAX1-SAG1 can significantly prolong survival of mice challenged with *T*. *gondii* RH strain, compared with the control. There was not an obvious prolonged survival of mice challenged with *T*. *gondii* RH strain when immunized with plasmid pVAX1-GRA1 or pVAX1-SAG1 alone. These results suggest that a combined GRA1 and SAG1 can enhance a protective effect against a lethal *T*. *gondii* infection, consistent with current studies (Li et al. 2010; Liu et al. 2009; Wang et al. 2009).

To explore the mechanisms of protective immunity induced by the DNA vaccine encoding GRA1 and SAG1, humoral and cellular immune responses were analyzed. For humoral immune responses, our data showed that the relative level of anti-*T*. *gondii* IgG antibody in mice immunized with pVAX1-GRA1 or pVAX1-SAG1 was elevated, comparing to controls, and it was further elevated by immunization with pVAX1-GRA1 + pVAX1-SAG1, suggesting that a DNA vaccine, encoding GRA1 and SAG1, could produce stronger humoral immunity.

It has been shown that protection against *T*. *gondii* is dependent on both CD4^+^ and CD8^+^ cells (Li et al. 2010). CD8^+^ T cells constitute the major T cell subset involved in acquired cellular immune protection against *T*. *gondii* (Tan et al. 2011). In vivo T cell depletion experiments indicate that CD8^+^ T cells are essential for survival of GRA1-vaccinated C3H mice during acute phase of *T*. *gondii* infection, while depletion of CD4^+^ T cells led to an increase in brain cyst burden during the chronic phase of infection (Scorza et al. 2003). We observed that 4 weeks after the last vaccination, the percentage of splenic CD8^+^ T cells in immunized mice increased in the pVAX1-GRA1 + pVAX1-SAG1 group, while pVAX1-GRA1- and pVAX1-SAG1-immunized mice did not exhibit any significant change. The CD4^+^/CD8^+^ ratio was reduced significantly. These data suggest that the immune protection against *T*. *gondii* challenge is elicited by multicomponent pVAX1-GRA1 + pVAX1-SAG1, and that it is mediated primarily through induction of CD8^+^ T subsets and a Th1-type cellular immune response.

NK cells are a kind of lymphocyte with powerful cytotoxicity and play an important role in innate immunity. We found that NK cell-killing activity of pVAX1-GRA1 + pVAX1-SAG1, pVAX1-SAG1, and pVAX1-GRA1 groups were all higher than that of controls, while the cytotoxicity of the pVAX1-GRA1 + pVAX1-SAG1 group was higher than that of pVAX1-GRA1 and pVAX1-SAG1, suggesting that combined GRA1 and SAG1 is more effective at enhancing NK cell activity.

Cytokines play an important role in Th cell function. Previous studies have shown that IFN-γ can promote Th1 differentiation, and that IL-4 can induce development of Th2 cells (Maggi et al. 1992). Th1 cells can further stimulate macrophages and CTL through IFN-γ, while Th2 cells regulate B cell helper activities by IL-4. Furthermore, IFN-γ and IL-2 are important stimulatory cytokines involved in the protection against parasitic infection. T cells and NK cells are the primary producers of IFN-γ, which enhances NK cell-killing activity and activates mononuclear macrophages to kill *T*. *gondii* intracellularly. IL-2 can activate the cytotoxic activity of T cells and facilitate production of various cytokines, such as IFN-γ. IL-2 also stimulates NK cell cytotoxicity, while IL-4 is thought to downregulate the inflammatory immune response (Matowicka-Karna et al. 2009; Suzuki et al. 1989, 1988). To evaluate the effects of vaccine pVAX1-GRA1 and pVAX1-SAG1, three cytokines were monitored. Our results indicate that pVAX1-GRA1 and pVAX1-SAG1 are capable of stimulating high levels of IFN-γ and IL-2, although IL-4 levels were similar across all groups, suggesting that vaccination was preferentially driving a Th1-type response. The empty pVAX1 plasmid appeared to also stimulate IFN-γ secretion, suggesting that the DNA macromolecule itself may function as an immunological adjuvant and induce nonspecific stimulation on the immune system.

In summary, our results demonstrate that a multicomponent DNA vaccine, encoding *T*. *gondii* GRA1 and SAG1, primes the immune system to generate a stronger humoral and cellular immune response and enhances protection against a *T*. *gondii* challenge. This combination vaccine may, therefore, provide a more effective means to combat troublesome *T*. *gondii* infections.

## References

[CR1] Alarcon JB, Waine GW, McManus DP (1999). DNA vaccines: technology and application as anti-parasite and anti-microbial agents. Adv Parasitol.

[CR2] Bhopale GM (2003). Development of a vaccine for toxoplasmosis: current status. Microbes Infect.

[CR3] Bivas-Benita M, Laloup M (2003). Generation of Toxoplasma gondii GRA1 protein and DNA vaccine loaded chitosan particles: preparation, characterization, and preliminary in vivo studies. Int J Pharm.

[CR4] Calabrese KS, Tedesco RC (2008). Serum and aqueous humour cytokine response and histopathological alterations during ocular Toxoplasma gondii infection in C57BL/6 mice. Micron.

[CR5] Cesbron-Delauw MF (1994). Dense-granule organelles of *Toxoplasma gondii*: their role in the host–parasite relationship. Parasitol Today.

[CR6] Couper KN, Nielsen HV (2003). DNA vaccination with the immunodominant tachyzoite surface antigen (SAG-1) protects against adult acquired Toxoplasma gondii infection but does not prevent maternofoetal transmission. Vaccine.

[CR7] Dautu G, Munyaka B, Carmen G (2007). *Toxoplasma gondii*: DNA vaccination with genes encoding antigens MIC2, M2AP, AMA1 and BAG1 and evaluation of their immunogenic potential. Exp Parasitol.

[CR8] Dubey JP (1990). Status of toxoplasmosis in sheep and goats in the United States. J Am Vet Med Assoc.

[CR9] Fachado A, Rodriguez A (2003). Protective effect of a naked DNA vaccine cocktail against lethal toxoplasmosis in mice. Vaccine.

[CR10] Fang R, Nie H, Wang Z (2009). Protective immune response in BALB/c mice induced by a suicidal DNA vaccine of the MIC3 gene of *Toxoplasma gondii*. Vet Parasitol.

[CR11] Jongert E, de Craeye S, Dewit J (2007). GRA7 provides protective immunity in cocktail DNA vaccines against *Toxoplasma gondii*. Parasite Immunol.

[CR12] Kato M, Claveria FG (2005). Toxoplasma gondii antigens GRA1 (p24) and SAG1 (p30): a comparison of their stimulatory influence on T-cell activation and cytokine expression in in vitro cultures. Pathobiology.

[CR13] Khosroshahi KH, Ghaffarifar F, Sharifi Z et al (2012) Comparing the effect of IL-12 genetic adjuvant and alum non-genetic adjuvant on the efficiency of the cocktail DNA vaccine containing plasmids encoding SAG-1 and ROP-2 of *Toxoplasma gondii*. Parasitol Res. doi:10.1007/s00436-012-2852-7 10.1007/s00436-012-2852-7 22350714

[CR14] Li B, Oledzka G, McFarlane RG (2010). Immunological response of sheep to injections of plasmids encoding *Toxoplasma gondii* SAG1 and ROP1 genes. Parasite Immunol.

[CR15] Li WS, Chen QX, Ye JX (2011). Comparative evaluation of immunization with recombinant protein and plasmid DNA vaccines of fusion antigen ROP2 and SAG1 from *Toxoplasma gondii* in mice: cellular and humoral immune responses. Parasitol Res.

[CR16] Liu S, Shi L, Cheng YB (2009). Evaluation of protective effect of multi-epitope DNA vaccine encoding six antigen segments of *Toxoplasma gondii* in mice. Parasitol Res.

[CR17] Liu MM, Yuan ZG, Peng GH (2010). *Toxoplasma gondii* microneme protein 8 (MIC8) is a potential vaccine candidate against toxoplasmosis. Parasitol Res.

[CR18] Lin J, Lin X (2010). Toxoplasma gondii: expression of GRA1 gene in endoplasmic reticulum promotes both growth and adherence and modulates intracellular calcium release in macrophages. Exp Parasitol.

[CR19] Maggi E, Parronchi P, Manetti R (1992). Reciprocal regulatory effects of IFN-gamma and IL-4 on the in vitro development of human Th1 and Th2 clones. J Immunol.

[CR20] Matowicka-Karna J, Dymicka-Piekarska V (2009). Does Toxoplasma gondii infection affect the levels of IgE and cytokines (IL-5, IL-6, IL-10, IL-12, and TNF-alpha)?. Clin Dev Immunol.

[CR21] Mevelec MN, Bout D (2005). Evaluation of protective effect of DNA vaccination with genes encoding antigens GRA4 and SAG1 associated with GM-CSF plasmid, against acute, chronical and congenital toxoplasmosis in mice. Vaccine.

[CR22] Montoya JG, Remington JS (2008). Management of Toxoplasma gondii infection during pregnancy. Clin Infect Dis.

[CR23] Scorza T, D’Souza S (2003). A GRA1 DNA vaccine primes cytolytic CD8(+) T cells to control acute Toxoplasma gondii infection. Infect Immun.

[CR24] Suzuki Y, Orellana MA (1988). Interferon-gamma: the major mediator of resistance against Toxoplasma gondii. Science.

[CR25] Suzuki Y, Conley FK (1989). Importance of endogenous IFN-gamma for prevention of toxoplasmic encephalitis in mice. J Immunol.

[CR26] Tan F, Hu X, Luo FJ (2011). Induction of protective Th1 immune responses in mice by vaccination with recombinant *Toxoplasma gondii* nucleoside triphosphate hydrolase-II. Vaccine.

[CR27] Vercammen M, Scorza T (2000). DNA vaccination with genes encoding Toxoplasma gondii antigens GRA1, GRA7, and ROP2 induces partially protective immunity against lethal challenge in mice. Infect Immun.

[CR28] Wang H, He S, Yao Y (2009). *Toxoplasma gondii*: protective effect of an intranasal SAG1 and MIC4 DNA vaccine in mice. Exp Parasitol.

[CR29] Xue M, He S, Cui Y (2008). Evaluation of the immune response elicited by multi-antigenic DNA vaccine expressing SAG1, ROP2 and GRA2 against *Toxoplasma gondii*. Parasitol Int.

[CR30] Zhang J, He S, Jiang H (2007). Evaluation of the immune response induced by multiantigenic DNA vaccine encoding SAG1 and ROP2 of *Toxoplasma gondii* and the adjuvant properties of murine interleukin-12 plasmid in BALB/c mice. Parasitol Res.

